# Atomic structure of the apoptosome: mechanism of cytochrome *c*- and dATP-mediated activation of Apaf-1

**DOI:** 10.1101/gad.272278.115

**Published:** 2015-11-15

**Authors:** Mengying Zhou, Yini Li, Qi Hu, Xiao-chen Bai, Weiyun Huang, Chuangye Yan, Sjors H.W. Scheres, Yigong Shi

**Affiliations:** 1 Ministry of Education Protein Science Laboratory, Center for Structural Biology, Tsinghua-Peking Center for Life Sciences, School of Life Sciences, School of Medicine, Tsinghua University, Beijing 100084, China;; 2 MRC Laboratory of Molecular Biology, Cambridge Biomedical Campus, Cambridge CB2 0QH, United Kingdom

**Keywords:** apoptosome, cryo-EM structure, apoptosis, Apaf-1, caspase activation, caspase-9

## Abstract

The apoptotic protease-activating factor 1 (Apaf-1) controls the onset of many known forms of intrinsic apoptosis in mammals. Apaf-1 exists in normal cells as an autoinhibited monomer. Upon binding to cytochrome *c* and dATP, Apaf-1 oligomerizes into a heptameric complex known as the apoptosome, which recruits and activates cell-killing caspases. Here we present an atomic structure of an intact mammalian apoptosome at 3.8 Å resolution, determined by single-particle, cryo-electron microscopy (cryo-EM). Structural analysis, together with structure-guided biochemical characterization, uncovered how cytochrome *c* releases the autoinhibition of Apaf-1 through specific interactions with the WD40 repeats. Structural comparison with autoinhibited Apaf-1 revealed how dATP binding triggers a set of conformational changes that results in the formation of the apoptosome. Together, these results constitute the molecular mechanism of cytochrome *c*- and dATP-mediated activation of Apaf-1.

## Abstract (precis)

In this study, Zhou et al. report the first atomic structure of the mammalian apoptosome, determined at 3.8 Å resolution by cryo-electron microscopy. These findings provide novel insight into how CytC relieves the autoinhibition of Apaf-1 and how dATP triggers Apaf-1 oligomerization.

Programmed cell death (apoptosis) is essential for the development and tissue homeostasis of all multicellular organisms (Horvitz 2003; Danial and Korsmeyer 2004). Apoptosis is executed by sequential activation of initiator and effector caspases (Shi 2002; Yan and Shi 2005). The major function of effector caspases, exemplified by the mammalian caspase-3, is to kill a cell by inflicting numerous cleavages on life-sustaining proteins. The primary role of an initiator caspase, on the other hand, is to cleave and hence activate specific effector caspases. In mammalian cells, the initiator caspase-9 is responsible for the activation of caspase-3. The autocatalytic activation of an initiator caspase depends critically on a specific, multiprotein complex. For caspase-9, this multiprotein complex is known as the apoptosome, which comprises seven copies of a heterodimer between apoptotic protease-activating factor 1 (Apaf-1) and cytochrome *c* (CytC) (Chai and Shi 2014). Because caspase-9 is essential for most known forms of intrinsic apoptosis, elucidation of its activation mechanism has been a central task toward mechanistic understanding of programmed cell death. The first major step toward this goal is to elucidate the mechanism of apoptosome assembly.

In normal mammalian cells, Apaf-1 exists as an ADP-bound, autoinhibited monomer. In response to various forms of intrinsic cell death stimuli, CytC is released from mitochondria into the cytoplasm (Liu et al. 1996), where CytC binds the monomeric Apaf-1 and primes it for oligomerization (Li et al. 1997; Zou et al. 1997). The replacement of ADP by dATP or ATP results in marked conformational changes, allowing Apaf-1 to form an activated, heptameric apoptosome (Acehan et al. 2002; Kim et al. 2005; Bao et al. 2007). Only the activated apoptosome is capable of facilitating the autocatalytic activation of caspase-9 (Rodriguez and Lazebnik 1999; Saleh et al. 1999; Zou et al. 1999). How does CytC interact with Apaf-1? How can these interactions facilitate nucleotide exchange and oligomerization of Apaf-1? What is the mechanism of apoptosome-mediated activation of caspase-9? Despite rigorous investigations, these questions have remained largely enigmatic.

The cryo-electron microscopy (cryo-EM) structures of the Apaf-1 apoptosome have been elucidated at a resolution range between 9.5 and 21 Å (Acehan et al. 2002; Yu et al. 2005; Yuan et al. 2010, 2011). These structures allowed placement of individual domains but failed to reveal specific interactions that govern the function of the apoptosome. X-ray structures of the monomeric, ADP-bound Apaf-1 provided an atomic view of the underpinnings of Apaf-1 autoinhibition (Riedl et al. 2005; Reubold et al. 2011). Together, these structural observations allowed the proposition of a speculative model that delineates the assembly of the apoptosome (Yuan et al. 2013). This model suffers from relatively low resolutions of prior EM structures and lack of supporting biochemical data.

In this study, we report the three-dimensional (3D) structure of an intact Apaf-1 apoptosome at a near-atomic resolution of 3.8 Å, determined by single-particle, cryo-EM analysis. We present results of structure-guided biochemical analyses. These experimental data give rise to a mechanistic pathway of Apaf-1 activation and apoptosome assembly.

## Results

### Overall structure of the Apaf-1 apoptosome

The full-length human Apaf-1 was expressed in baculovirus-infected insect cells and biochemically purified to homogeneity. Assembly of an intact apoptosome was completed through incubation of the purified Apaf-1 protein with an excess amount of horse CytC and 1 mM dATP. The assembled apoptosome exhibited excellent solution behavior on gel filtration (Supplemental Fig. S1A) and markedly stimulated the proteolytic activity of caspase-9. We imaged the apoptosome sample under cryo-conditions on an FEI Titan Krios microscope operating at 300 kV and collected 912 micrographs (Supplemental Fig. S1B). We chose a total of 202,932 individual particles for reference-free two-dimensional (2D) classification (Supplemental Fig. S1C). After 3D classification, a subset of 134,919 particles was used for image construction, giving a final overall resolution of 3.8 Å on the basis of the gold Fourier shell correlation (FSC) standard (Supplemental Figs. S1D, S2).

**Figure 1.**
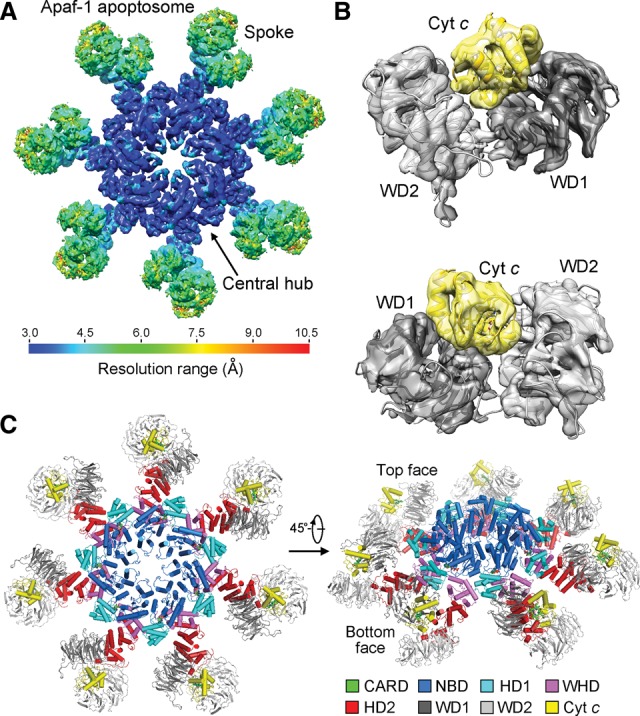
Overall structure of the Apaf-1 apoptosome. (*A*) An overall view of the EM density for the Apaf-1 apoptosome. The resolution is color-coded for different regions of the apoptosome. The surface view of the apoptosome is shown here. The resolution goes to 3.0–3.5 Å in the central hub of the apoptosome. (*B*) Two close-up views of the EM density surrounding CytC. The two β propellers WD1 and WD2 consist of WD40 repeats 1–7 and 8–15, respectively. (*C*) Overall structure of the Apaf-1 apoptosome. Two views are shown. The top face refers to the CytC-exposed side of the apoptosome disk. CytC is colored yellow, and the domains within each Apaf-1 protomer are color-coded. *A* and *B* were prepared using Chimera (Pettersen et al. 2004). Except for Figures 5 and 6, all other figures were prepared using PyMol (http://www.pymol.org).

The overall architecture of the Apaf-1 apoptosome is similar to that reported previously (Yuan et al. 2013), comprising a central hub and seven spokes (Fig. 1A). The local resolution for the vast majority of the central hub ranges between 3.0 and 3.5 Å (Fig. 1A), which represents a qualitative improvement over previously reported resolutions and, for the first time, allows assignment of specific interactions involving amino acid side chains in the activated apoptosome (Supplemental Fig. S2). The local resolution at the spokes is considerably lower (Fig. 1A); local masking strategy markedly improved the resolution in this region to ∼5 Å, which allows unambiguous determination of the interface between Apaf-1 and CytC (Fig. 1B).

Apaf-1 contains a caspase recruitment domain (CARD) at the N terminus, a nucleotide-binding domain (NBD), a helical domain (HD1), a winged helix domain (WHD), a second helical domain (HD2), and 15 WD40 repeats at the C-terminal half. The central hub consists of seven copies each of the NBD, HD1, and WHD (Fig. 1C). The 15 WD40 repeats in each spoke constitute two β propellers named WD1 and WD2, which are connected to the central hub through the HD2 domain. The seven CARDs exhibit no EM density, likely reflecting their dynamic locations. CytC is sandwiched by the front faces of the two β propellers. The CytC-exposed side of the apoptosome is hereafter referred to as the top face (Fig. 1C), which is characterized by a ring of positively charged residues at the surface of the central hub (Supplemental Figs. S3, S4). In contrast, the bottom face of the apoptosome is enriched by negatively charged amino acids (Supplemental Figs. S3, S4).

Compared with the reported structure of the apoptosome at 9.5 Å resolution (Yuan et al. 2010), our structure at 3.8 Å reveals a number of novel findings. First, the observed docking interface between the two β propellers and CytC in our structure is different from that proposed previously (Yuan et al. 2010, 2013). Compared with the published model (Yuan et al. 2010, 2013), CytC undergoes a rotation of ∼90° (Supplemental Fig. S5). This structural revelation has important ramifications for understanding how CytC releases the autoinhibition of the monomeric Apaf-1. Second, nucleotide binding is observed for the first time in the activated Apaf-1 apoptosome, and dATP is coordinated by several key residues through a number of specific hydrogen bonds (H bonds; discussed later). Third and most importantly, the markedly improved resolution allows visualization of atomic features and specific interactions, which govern Apaf-1 activation and oligomerization.

### Intramolecular domain stacking

**Figure 2.**
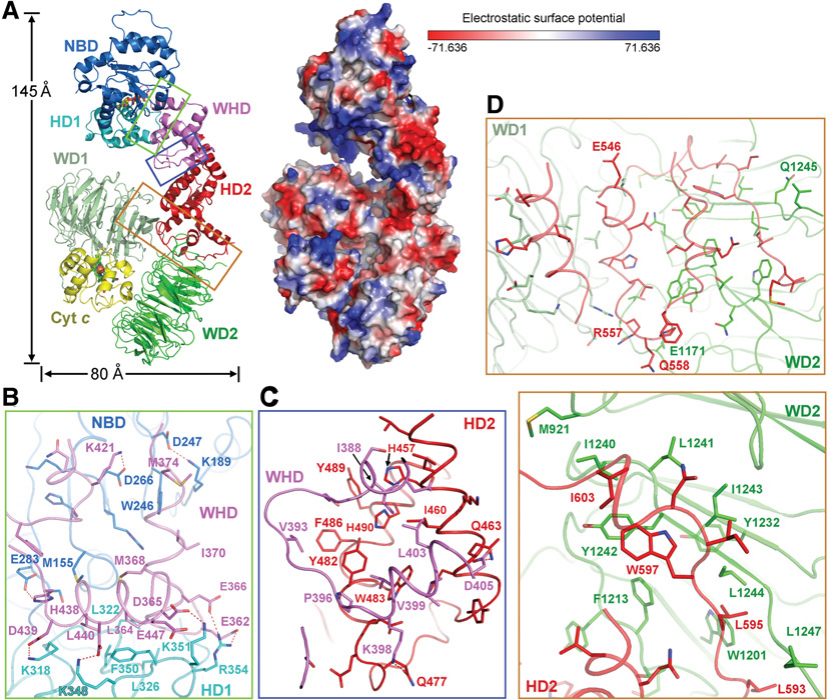
Structure of an activated Apaf-1 protomer in the apoptosome. (*A*) Structure of an activated Apaf-1 protomer in the apoptosome is shown in cartoon representation (*left* panel) and electrostatic surface potential (*right* panel). Three boxed interdomain interfaces are detailed in *B*–*D*. (*B*) A close-up view of the interface between WHD and the NBD–HD1 module. Potential hydrogen bonds are represented by red dashed lines. (*C*) A close-up view of the interface between WHD and HD2. This interface contains a large number of van der Waals contacts. (*D*) A close-up view of the interface between HD2 and the two β propellers. The intensity of interaction between HD2 and WD2 is considerably stronger than that between HD2 and WD1. The *bottom* panel zooms in on the van der Waals interactions mediated by three hydrophobic residues from HD2: Leu595, Trp597, and Ile603.

Our cryo-EM structure of the apoptosome represents the first experimentally determined atomic model of the activated Apaf-1, which exhibits a dimension of ∼145 Å in height, 80 Å in width, and 55 Å in thickness (Fig. 2A). The overall appearance of Apaf-1 resembles a seahorse, with NBD/HD1 and WD1/WD2/CytC corresponding to the head and the tail, respectively (Fig. 2A). The head and the tail are spatially separated by a gap of 12–14 Å and are connected by the WHD and HD2 domains. The surface of Apaf-1 is enriched by charged amino acids (Fig. 2A), which engender specific intermolecular H bonds and salt bridges between neighboring Apaf-1 protomers in the apoptosome (discussed later). The extended structure of Apaf-1 is held together through extensive interdomain interactions, mainly involving three interfaces: between WHD and NBD/HD1 (Fig. 2B), between WHD and HD2 (Fig. 2C), and between HD2 and WD1/WD2 (Fig. 2D).

The WHD stacks against the NBD/HD1 module through a combination of H bonds and van der Waals contacts (Fig. 2B). The periphery contains at least eight potential H bonds, exemplified by four pairs of aspartate and lysine residues. The charged side chains of Asp365, Lys421, Asp439, and Asp443 from WHD interact with the side chains of Lys351, Asp266, Lys318, and Lys348, respectively. At the center of this interface, the hydrophobic side chains of Leu364, Met368, and Leu440 from WHD are nestled in a shallow hydrophobic pocket formed by side chains from Met155/Ala156 from NBD and Leu322/Val323/Leu326/Phe350 from HD1 (Fig. 2B). In contrast to the interface between WHD and NBD/HD1, WHD interacts with HD2 through predominantly van der Waals contacts (Fig. 2C). Notably, Ile388, Val393, Val399, and Leu403 from WHD closely stack against a hydrophobic surface patch formed by His457, Ile460, His490, and four aromatic residues: Tyr482, Trp483, Phe486, and Tyr489.

The interface between HD2 and the WD1/WD2 module involves a large buried surface area of ∼3362 Å^2^, of which approximately two-thirds are contributed by the WD2 propeller (Fig. 2D, top panel). In particular, four hydrophobic residues—Leu593, Leu595, Trp597, and Ile603—from HD2 follow a hydrophobic corridor on the surface of WD2 that is formed by 11 uncharged residues (Fig. 2D, bottom panel). This high density of van der Waals contacts between HD2 and WD2 is reminiscent of that between WHD and HD2 but contrasts the relatively sparse interactions between HD2 and WD1. This analysis is fully consistent with the notion that the three domains WHD, HD2, and WD2 form a rigid rod (Reubold et al. 2011). The WHD/HD2/WD2 rod remains unchanged in response to Apaf-1 activation (discussed later).

### Interactions among adjacent Apaf-1 protomers in the apoptosome

**Figure 3.**
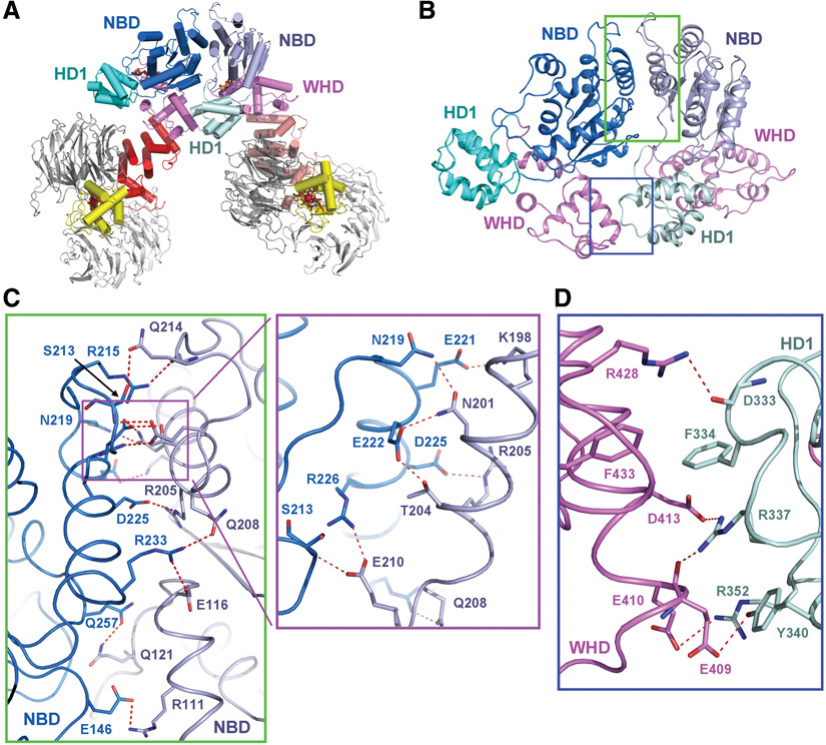
Interactions between two adjacent Apaf-1 protomers in the apoptosome. (*A*) An overall view of two adjacent Apaf-1 protomers in the apoptosome. The top face is shown. The domains are color-coded. (*B*) A focused view of the domains that mediate intermolecular interactions. An Apaf-1 protomer uses its NBD and WHD to contact the NBD and HD1, respectively, of an adjacent Apaf-1 protomer. The interactions are detailed in *C* and *D*. (*C*) A close-up view of the interface between two neighboring NBDs. This interface contains a large number of H-bonds (red dashed lines). An *inset* is shown to highlight a network of closely spaced H bonds. (*D*) A close-up view of the interface between the WHD of one Apaf-1 protomer and HD1 of an adjacent Apaf-1 protomer.

In the apoptosome, two adjacent Apaf-1 protomers interact with each other exclusively through their N-terminal regions (Fig. 3A). Specifically, the NBDs and WHDs of one protomer stack closely against the NBD and HD1, respectively, of an adjacent protomer (Fig. 3B). This mode of interaction, characterized by a prominent role of the WHD, is uniquely shared among the NOD family of proteins (Qi et al. 2010; Pang et al. 2015) and differs from that for most other AAA^+^ motors (Diemand and Lupas 2006; Erzberger and Berger 2006). Our cryo-EM structure at 3.8 Å resolution allows identification of interprotomer interactions that govern the assembly of the activated apoptosome.

The stacking interactions between two adjacent Apaf-1 protomers involve a buried surface area of ∼2805 Å^2^, of which three-quarters are contributed by the interface between two neighboring NBDs. This interface is prominently configured by 13 potential H bonds (Fig. 3C). At the center of the interface, the carboxylate side chain of Asp225 accepts an intermolecular H bond from the guanidinium group of Arg205, whereas the guanidinium group of Arg233 donates two intermolecular H bonds to Glu116 and Gln208. At one side of the periphery, Glu146 and Gln257 each accept an intermolecular H bond from Arg111 and Gln121, respectively. At the other side, a network of eight H bonds is placed close to each other and may play a key role in stabilizing the interface (Fig. 3C).

The interface between the WHD of one Apaf-1 protomer and HD1 of an adjacent protomer is also enriched by charged amino acids (Fig. 3D) but appears to play a supporting role for apoptosome assembly, as judged by a much smaller buried surface area of ∼763 Å^2^ compared with that between two adjacent NBDs. Consistent with this analysis, a truncated Apaf-1 without the WHD still retained the ability to form an oligomer (Hu et al. 1998; Srinivasula et al. 1998). This interface features five intermolecular H bonds (exemplified by a bifurcated contact from Arg337 of HD1) and van der Waals interactions between Phe433 and Phe334 (Fig. 3D).

The intermolecular interactions among neighboring Apaf-1 protomers comprise mostly charge-stabilized H bonds. This structural observation sharply contrasts the interdomain interactions within a single Apaf-1 protomer, which are dominated by van der Waals contacts (Fig. 2). Compared with hydrophobic interactions, H bonds and salt bridges usually exhibit faster kinetics and thus are more amenable to biological regulation—in this case, apoptosome assembly and disassembly for the activation of caspase-9. Therefore, the atomic interactions both within an Apaf-1 protomer and between adjacent protomers appear to have evolved to facilitate the underlying functions.

### Recognition of CytC by Apaf-1

**Figure 4.**
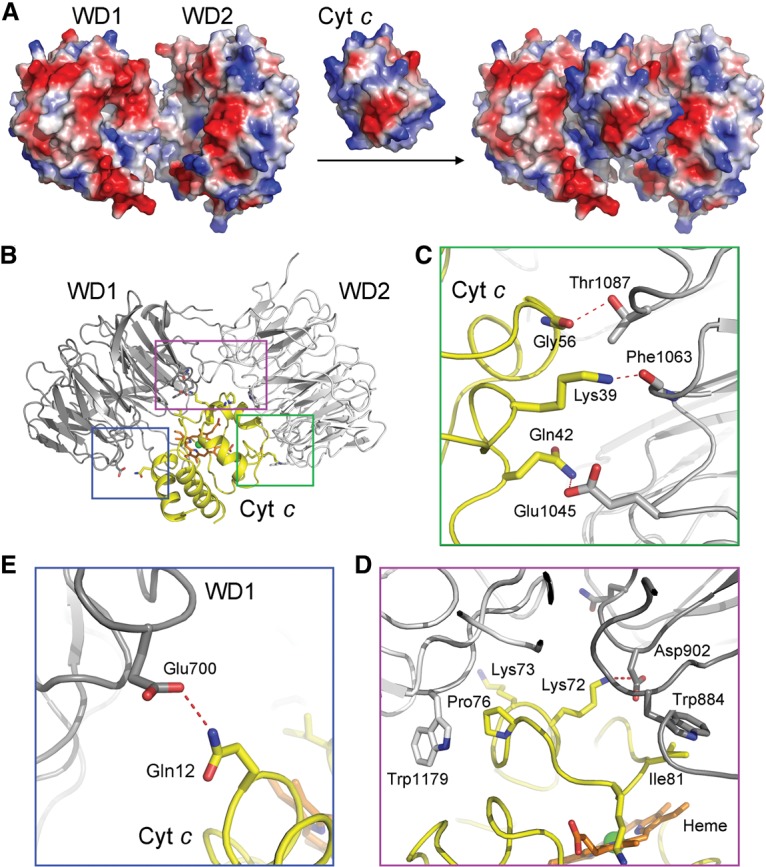
Recognition of CytC by the WD1 and WD2 domains of Apaf-1. (*A*) CytC is sandwiched between WD1 and WD2 with excellent complementarity in shape and charge. CytC and WD1/WD2 are shown in electrostatic surface potential. (*B*) A schematic diagram of the interactions between CytC and the WD1/WD2 domains. The three boxed regions are detailed in *C*–*E*. (*C*) A close-up view of the interface between CytC and WD2. This interface contains three putative intermolecular H bonds (red dashed lines). Notably, Gly56 contributes a H bond and makes van der Waals contacts to WD2. (*D*) A close-up view of the interface between CytC and the adjoining area of WD2 and WD1. Pro76 and Ile81 make van der Waals contacts to neighboring structural elements from WD2 and WD1, respectively. Lys72 donates a H bond to Asp902 of WD1. (*E*) A close-up view of the interface between CytC and WD1. Gln12 of CytC likely makes a H bond to Glu700 of WD1.

In contrast to the central hub, the periphery of the Apaf-1 apoptosome, comprising the WD40 repeats and CytC, displays relatively low resolution. This feature may reflect the inherent property of the apoptosome: rigid conformation at the central hub and relatively mobile nature in the spokes. The strategy of applying local masks helped improve the EM density and allowed unambiguous determination of CytC binding. CytC is sandwiched between the two front faces of WD1 and WD2, with excellent shape and charge complementarity (Fig. 4A). Although the secondary structural elements in CytC and WD1/WD2 and their relative orientation are well defined (Fig. 1B), the improved EM density is inadequate for assignment of specific side chains. Nonetheless, we manually modeled putative interactions for specific residues between CytC and the WD1–WD2 module in Apaf-1.

The interface between CytC and WD2 of Apaf-1, involving ∼1242 Å^2^ buried surface area, appears to contain more specific interactions than that between CytC and WD1, which entails ∼712 Å^2^ buried surface area (Fig. 4B). The interface between CytC and WD2 of Apaf-1 contains three putative intermolecular H bonds and a number of van der Waals contacts. In particular, Gly56 of CytC is located in close proximity to Thr1087 from WD2, with a H bond between the side chain of Thr1087 and the carbonyl oxygen of Gly56 (Fig. 4C). The side chains of Lys39 and Gln42 of CytC may donate two H bonds to the carbonyl oxygen of Phe1063 and the side chain of Glu1045, respectively. In addition, Pro76 of CytC makes direct van der Waals contacts to the indole ring of Trp1179 from WD2 of Apaf-1 (Fig. 4D). The interface between CytC and WD1 of Apaf-1 contains only one putative H bond between Gln12 of CytC and Glu700 from WD1 (Fig. 4E). Most notably, however, Ile81 of CytC is in close contact with Trp884 from WD1 (Fig. 4D). Lys72 of CytC, which had been previously implicated in playing a role in binding to Apaf-1 (Yu et al. 2001), is likely H-bonded to Asp902 from WD1 (Fig. 4D).

**Figure 5.**
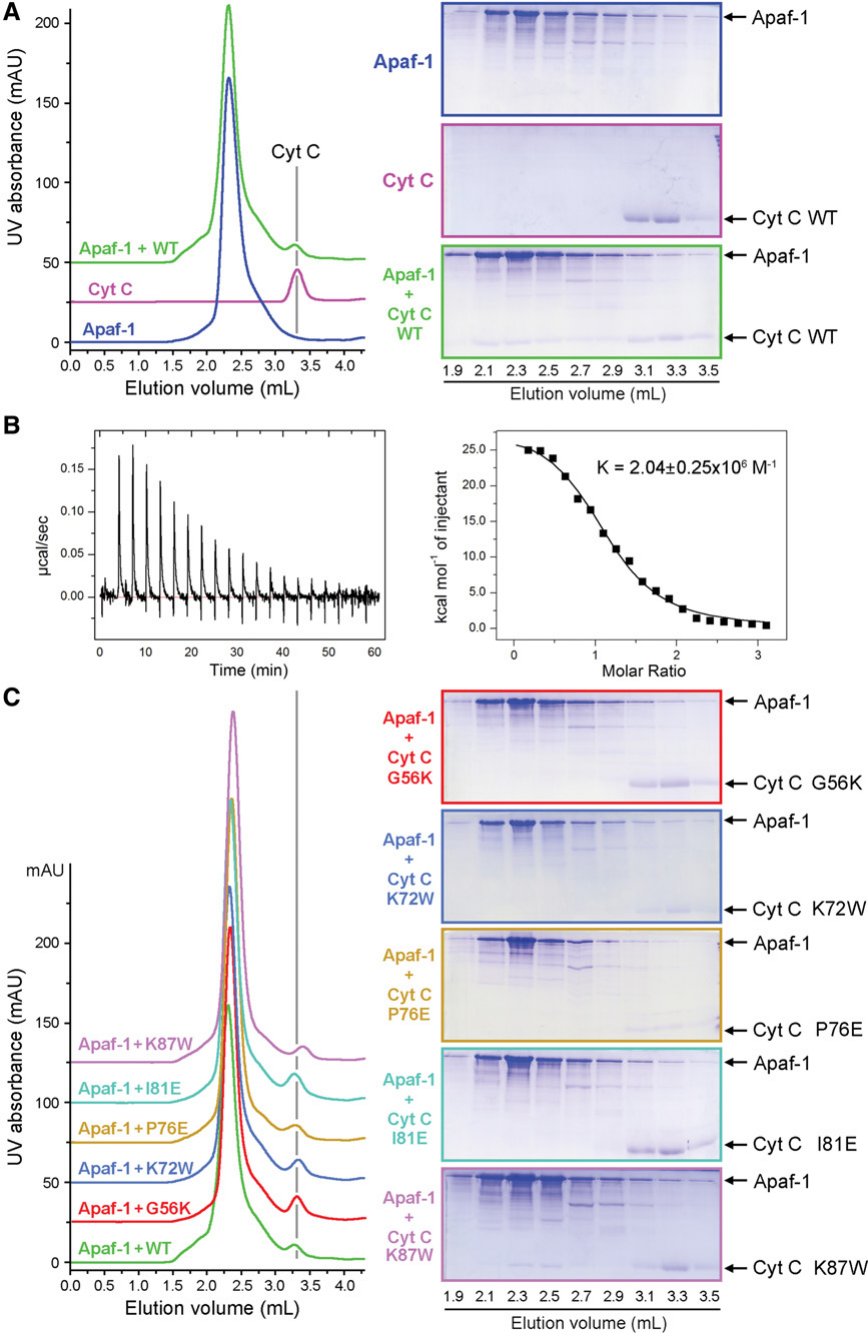
Biochemical characterization of the interactions between Apaf-1 and CytC. (*A*) Wild-type CytC forms a stable complex with the full-length Apaf-1. Three gel filtration chromatograms are shown in the *left* panel, and the corresponding fractions from gel filtration are shown on SDS-PAGE gels in the *right* panels. To clearly visualize the results, the baselines for the magenta (CytC alone) and green (Apaf-1 + wild-type CytC) chromatograms were shifted by 25 and 50 mAU, respectively. (*B*) Wild-type CytC binds to the full-length Apaf-1 with an association constant of ∼2.04 × 10^6^ M^−1^ ± 0.25 × 10^6^ M^−1^, which corresponds to a binding affinity of ∼0.49 µM ± 0.06 µM. Shown here are the raw data from ITC (*left* panel) and curve fitting (*right* panel). (*C*) Assessment of the interactions between five representative CytC mutants and Apaf-1 by gel filtration. To better display the results, the baselines for the five CytC mutants were shifted incrementally by 25 mAU each. Only wild-type CytC and the K87W mutant retained stable association with Apaf-1.

To corroborate the structural observations, we generated five representative missense mutations in CytC, individually purified these mutant proteins to homogeneity, and examined their abilities to associate with Apaf-1. Free wild-type CytC exhibited an elution volume of ∼3.3 mL on gel filtration (Fig. 5A). Upon incubation of the full-length human Apaf-1 (residues 1–1248) with an excess amount of wild-type CytC, the elution volume for a fraction of wild-type CytC was shifted to ∼2.3 mL, which coincides with that of Apaf-1 (Fig. 5A). This observation suggests that wild-type CytC formed a stable complex with Apaf-1. This conclusion was confirmed by isothermal titration calorimetry (ITC), which revealed a dissociation constant of ∼0.49 µM ± 0.06 µM for the Apaf-1–CytC interaction (Fig. 5B). In contrast to wild-type CytC, four CytC mutants—G56K, K72W, P76E, and I81E—abolished stable association with Apaf-1, as judged by gel filtration analysis (Fig. 5C) as well as ITC (Supplemental Fig. S6). These results nicely corroborate the structural finding that Gly56, Lys72, Pro76, and Ile81 all play an important role in stabilizing the interface between CytC and Apaf-1 (Fig. 4C,D). Lys87 of CytC makes no direct contact to WD1 or WD2; consequently, the mutation K87W in CytC had little impact on its association with Apaf-1 (Fig. 5C; Supplemental Fig. 6).

### Impact of CytC mutations on caspase-9 activation

**Figure 6.**
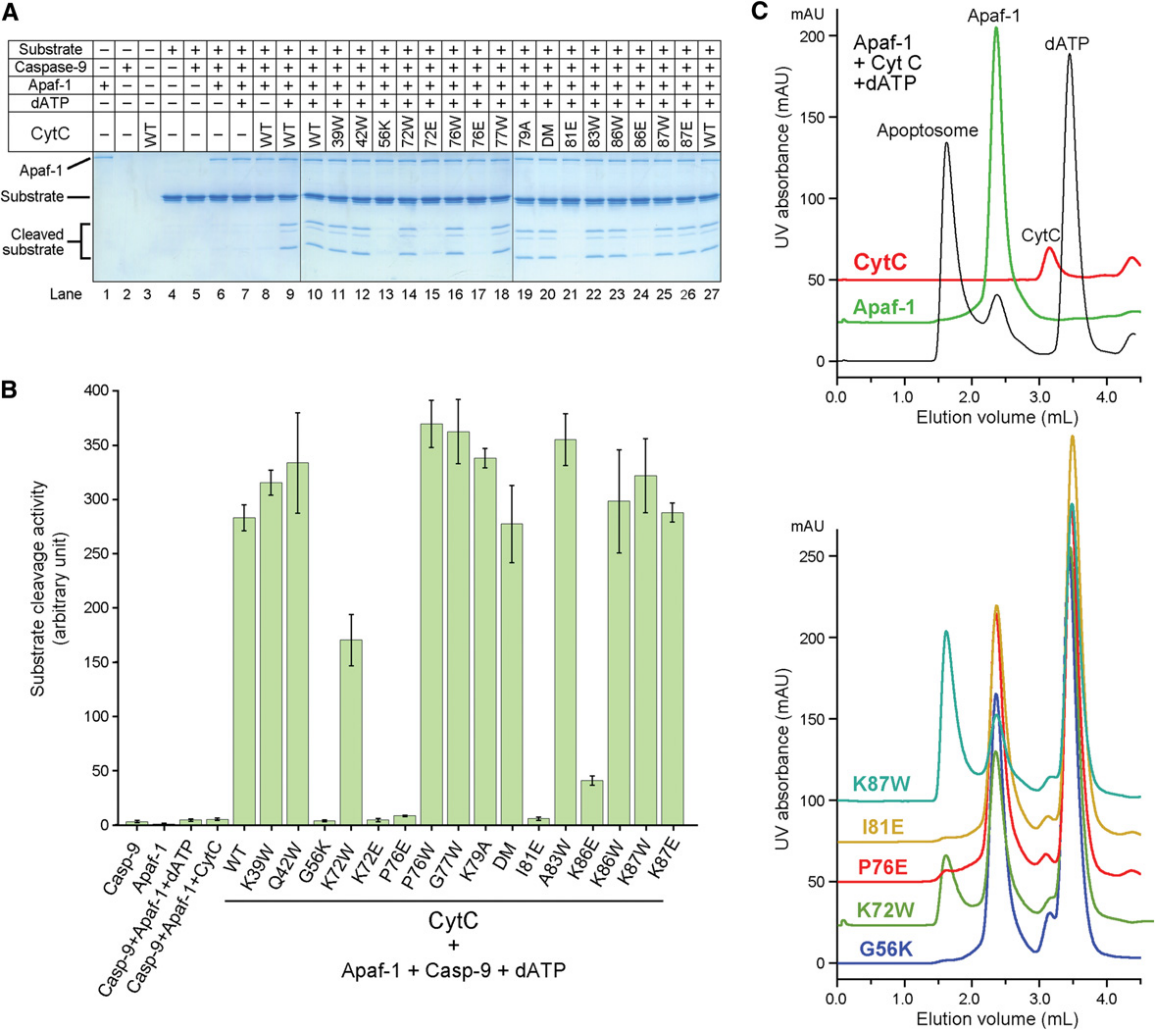
Impact of CytC mutations on apoptosome assembly and caspase-9 activation. (*A*) Impact of CytC mutations on caspase-9 activation in an in vitro cleavage assay using caspase-3 (C163A) as the substrate. For each CytC mutant, only the residue number and the target residue are labeled. For example, “56K” denotes G56K. The only double mutation (DM) denotes K79F and I81W. (*B*) Impact of these CytC mutations on caspase-9 activation in a second in vitro cleavage assay using the fluorogenic peptide Ac-LEHD-AFC as the substrate. (*C*) Assessment of the impact of representative CytC mutations on apoptosome formation by gel filtration. The wild-type control is shown in the *top* panel, where the baselines for the green (Apaf-1 alone) and red (CytC alone) chromatograms were shifted by 25 and 50 mAU, respectively. The five representative CytC mutants are displayed in the *bottom* panel, where the baselines of the chromatograms were shifted incrementally by 25 mAU each. Notably, the CytC-K72W mutant partially retained the ability to form an apoptosome.

The association of CytC with Apaf-1 is thought to precede nucleotide exchange and apoptosome formation (Chai and Shi 2014). Mutations in CytC that weaken its interaction with Apaf-1 are predicted to have negative consequences on the function of apoptosome. To examine this scenario, we generated 11 additional missense mutations in CytC and investigated the ability of all 16 CytC mutants to activate caspase-9 using an in vitro proteolytic cleavage assay. In this assay, the catalytically inactive caspase-3 (C163A) protein, which is a physiological substrate of caspase-9, was employed as the substrate. In the absence of CytC, caspase-9 had little proteolytic activity toward the substrate (Fig. 6A, lanes 2,4–7). In the presence of Apaf-1 and dATP, wild-type CytC markedly stimulated the protease activity of caspase-9 (Fig. 6A, lane 9). In contrast, five CytC missense mutants (G56K, K72E, P76E, I81E, and K86E), each involving mutation to a charged amino acid, exhibited markedly decreased levels of protease activity (Fig. 6A, lanes 13,15,17,21,24). For three of the five targeted residues (Lys72, Pro76, and Lys86), mutation to Trp was also generated; intriguingly, all three mutants retained a similar level of protease activity compared with wild-type CytC (Fig. 6A, lanes 14,16,23). These observations suggest that alteration of the surface charge distribution in CytC, especially by introducing a charged amino acid into a generally hydrophobic environment (G56K, P76E, and I81E), is more detrimental than merely introducing a bulkier residue (Trp). Furthermore, these results indicate that the compromised Apaf-1 binding by CytC-K72W (Fig. 5C; Supplemental Fig. S6) had been alleviated in the presence of dATP. Other CytC mutants also retained a similar level of protease activity compared with wild-type CytC. Together, these experimental findings demonstrate that the residues in CytC that play a key role at the interface with Apaf-1 are indispensable for caspase-9 activation, presumably due to compromised formation of the apoptosome.

To further corroborate this conclusion, we switched to a more sensitive cleavage assay in which the caspase-3 substrate was replaced by the fluorogenic peptide Ac-LEHD-AFC (Fig. 6B). This assay allows convenient quantification of the protease activity and is more sensitive than that using the caspase-3 (C163A) protein as the substrate (Fig. 6A). Gratifyingly, the results are fully consistent with those obtained using caspase-3 (C163A). The presence of both wild-type CytC and dATP, but not a single agent alone, led to a drastically improved caspase-9 activity in the presence of Apaf-1. Consistently, wild-type CytC and Apaf-1 formed a stable apoptosome in the presence of 1 mM dATP (Fig. 6C, top panel). Compared with wild-type CytC, the positive control mutants exemplified by K87E and K87W stimulated caspase-9 activity to a similar level (Fig. 6B); the CytC mutant K87W also allowed efficient assembly of the apoptosome (Fig. 6C, bottom panel). In sharp contrast to wild-type CytC, the four mutants G56K, K72E, P76E, and I81E nearly abolished the ability to stimulate caspase-9 activity in the presence of Apaf-1 (Fig. 6B); accordingly, the CytC mutants G56K, P76E, and I81E abrogated apoptosome formation (Fig. 6C, bottom panel). The CytC mutant K72W retained the ability to stimulate caspase-9 activity (Fig. 6B) and supported apoptosome formation (Fig. 6C, bottom panel).

### Conformational changes and nucleotide binding

**Figure 7.**
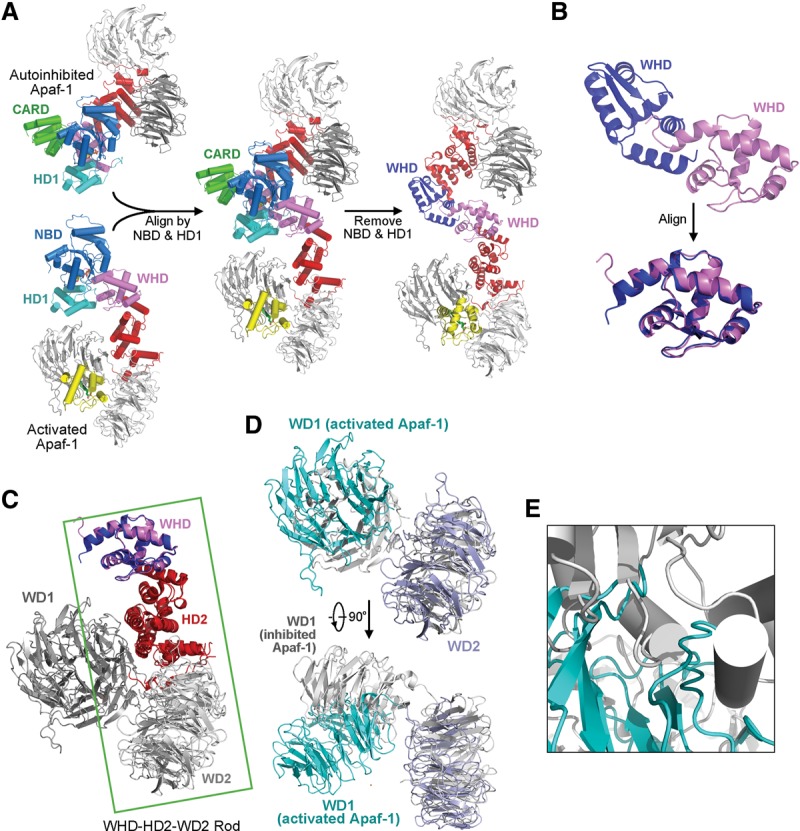
Conformational differences between the autoinhibited Apaf-1 and the activated Apaf-1 protomer from the apoptosome. (*A*) Structural overlay of the autoinhibited Apaf-1 and the activated Apaf-1 protomer on their respective NBD–HD1 modules. (*B*) A close-up view of the two WHDs derived from *A*. These two WHDs are related to each other by a pseudo-twofold symmetry axis. (*C*) Superposition of the two WHDs results in near-perfect alignment of the WHD–HD2–WD2 rods from the two Apaf-1 molecules. (*D*) A close-up view of the WD1 and WD2 derived from *C*. Relative to the autoinhibited Apaf-1, WD1 in the activated Apaf-1 promoter undergoes a rotation of ∼60° toward the WD2. This movement is triggered by CytC binding. (*E*) Movement of WD1 would cause severe steric clashes with the NBD in the autoinhibited conformation of Apaf-1. This finding explains why, in addition to WD1, CytC binding must induce conformational changes in other domains of Apaf-1.

Formation of the apoptosome requires drastic conformational rearrangements in Apaf-1. The relative orientation of NBD versus HD1 remains largely unchanged between the ADP-bound, autoinhibited Apaf-1 and the dATP-bound, activated Apaf-1. Structural alignment around the NBD–HD1 module reveals a pseudo-twofold rotation symmetry in the relative positioning of the WHD–HD2–WD2 rods (Fig. 7A), which is exemplified by the WHDs (Fig. 7B). Application of this rotation symmetry results in near-perfect registry between the two WHD–HD2–WD2 rods (Fig. 7C). In contrast to the WHD–HD2–WD2 rods, the two WD1 propellers are positioned quite differently in the two Apaf-1 molecules (Fig. 7D). Compared with that in the autoinhibited Apaf-1, the WD1 propeller in the activated Apaf-1 is rotated by ∼60° toward WD2 (Fig. 7D), which is apparently caused by CytC binding. Such a drastic conformational change in WD1 would be incompatible with the overall conformation of the autoinhibited Apaf-1 because WD1 would sterically clash with the NBD (Fig. 7E). This analysis strongly argues that CytC binding may facilitate the conformational changes in Apaf-1 that destabilize the autoinhibited state.

**Figure 8.**
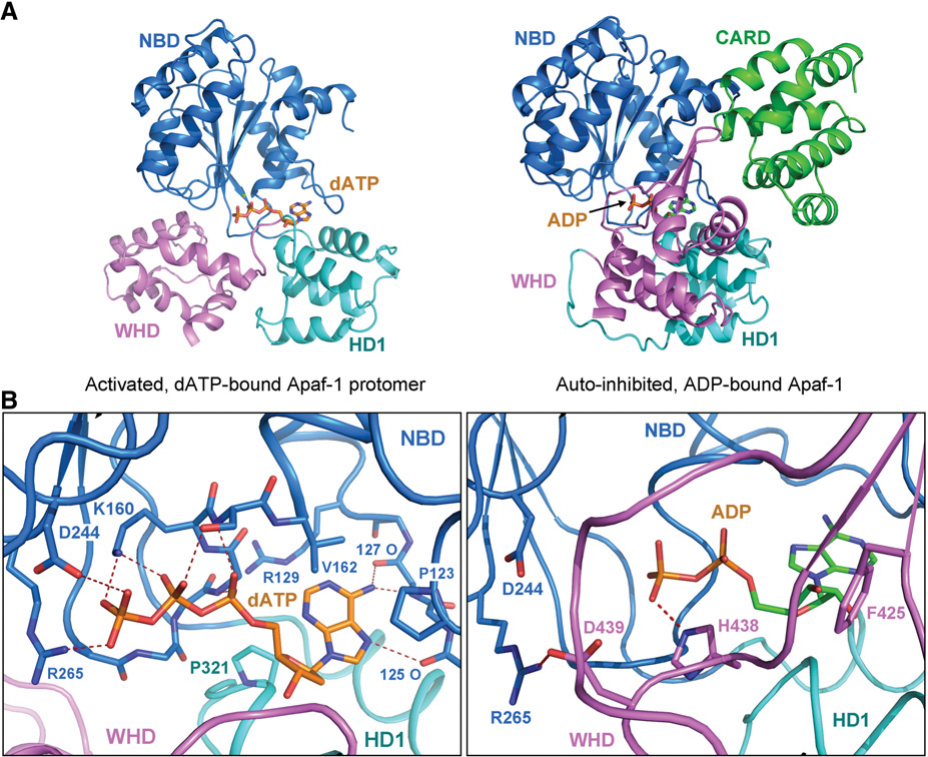
Comparison of nucleotide binding between the autoinhibited Apaf-1 and the activated Apaf-1 protomer from the apoptosome. (*A*) An overall comparison of the domains that contribute to nucleotide binding. (*Left* panel) In the activated Apaf-1 protomer, dATP is bound exclusively by NBD and HD1 and exposed to solvent. (*Right* panel) In the autoinhibited Apaf-1, ADP is buried and bound at the interface of NBD, HD1, and WHD. (*B*) A close-up comparison of nucleotide binding in these two Apaf-1 molecules. (*Left* panel) In the activated Apaf-1 protomer, dATP is recognized through a number of H bonds mediated by the Sensor I Arg265, the Walker B residue Asp244, the P-loop residue Lys160, and other main chain groups. No residue from WHD is involved in coordinating dATP. (*Right* panel) In the autoinhibited Apaf-1, ADP is recognized in part by the MHD/LHD residue His438 through a H bond.

Thus, the most prominent conformational switch during Apaf-1 activation involves repositioning of WHD relative to the NBD–HD1 module. Such a switch results in the dislocation of WHD and consequent exposure of a buried ADP molecule in the autoinhibited Apaf-1 to solvent in the activated Apaf-1 (Fig. 8A). In fact, the nucleotide-binding pocket in the autoinhibited Apaf-1 is sufficiently spacious to accommodate an ATP or dATP molecule, except that the binding pocket is blocked by the WHD. The solvent-exposed nature of the nucleotide-binding pocket in the activated Apaf-1 may greatly facilitate the displacement of ADP by ATP or dATP.

There are at least three prominent differences in nucleotide binding between the autoinhibited and activated Apaf-1 molecules (Fig. 8B). First, His438 of the MHD/LHD motif directly coordinates ADP in the autoinhibited Apaf-1 but is distant from the nucleotide-binding pocket in the activated Apaf-1. Second, the conserved Sensor I residue Arg265 among the AAA^+^ proteins is H-bonded to Asp439 in the autoinhibited Apaf-1 but directly coordinates dATP by donating a H bond to the γ-phosphate group (Fig. 8B). Third, the Walker B residue Asp244 is idle in the autoinhibited Apaf-1 but directly contacts the γ-phosphate group of dATP in the activated Apaf-1, perhaps as a consequence of realignment of Arg265. In addition, the phosphate groups of the bound dATP molecule in the activated Apaf-1 are recognized by a number of specific H bonds from Lys160 and other residues from the P loop (Fig. 8B). Similar to that in the autoinhibited Apaf-1 (Riedl et al. 2005; Reubold et al. 2011), the adenine base of dATP appears to be recognized by specific H bonds from main chain groups in the activated Apaf-1.

## Discussion

The concept of apoptosis and its role in physiology and health were elegantly described in 1972 by Kerr et al. (1972). Since then, this cellular suicide program has been subjected to rigorous investigation in several model organisms, exemplified by the identification of a linear pathway of programmed cell death in the nematode *Caenorhabditis elegans* (Horvitz 2003). The functions of the three *C. elegans* genes *ced-3*, *ced-4*, and *ced-9* were clearly delineated (Hengartner 1996), with CED-3 being a cell-killing protease known as caspase (Xue et al. 1996), CED-4 being a novel adaptor protein required for CED-3 activation (Yuan and Horvitz 1992), and CED-9 being a functional homolog of the mammalian oncogene Bcl-2 (Hengartner and Horvitz 1994). The discovery of Apaf-1 as a mammalian homolog of CED-4 filled a critical missing piece in the description of the conserved apoptosis pathway (Zou et al. 1997). More importantly, the identification of the mitochondrial protein CytC as a cytosolic activator of Apaf-1 provides unequivocal evidence for the concept that mitochondria may play an essential role in the apoptosis of mammalian cells (Liu et al. 1996; Li et al. 1997). CytC was subsequently found to work together with dATP/ATP in the assembly of the apoptosome (Li et al. 1997).

In the past two decades, numerous advances have been achieved in the biochemical and structural studies of apoptotic mechanisms in several model organisms (Fesik 2000; Yan and Shi 2005; Chai and Shi 2014). However, a number of important questions remain unanswered. The molecular mechanism of CytC release from the mitochondria is yet to be elucidated. Once in the cytoplasm, how CytC relieves the autoinhibited conformation of Apaf-1 and how CytC cooperates with dATP to facilitate apoptosome formation are also largely enigmatic. Addressing these latter questions requires 3D structural information of the Apaf-1 apoptosome at an atomic resolution, which has remained elusive despite repeated efforts. In this study, we report the cryo-EM structure of an intact Apaf-1 apoptosome at 3.8 Å resolution, which reveals the precise inner workings of a mammalian apoptosome.

**Figure 9.**
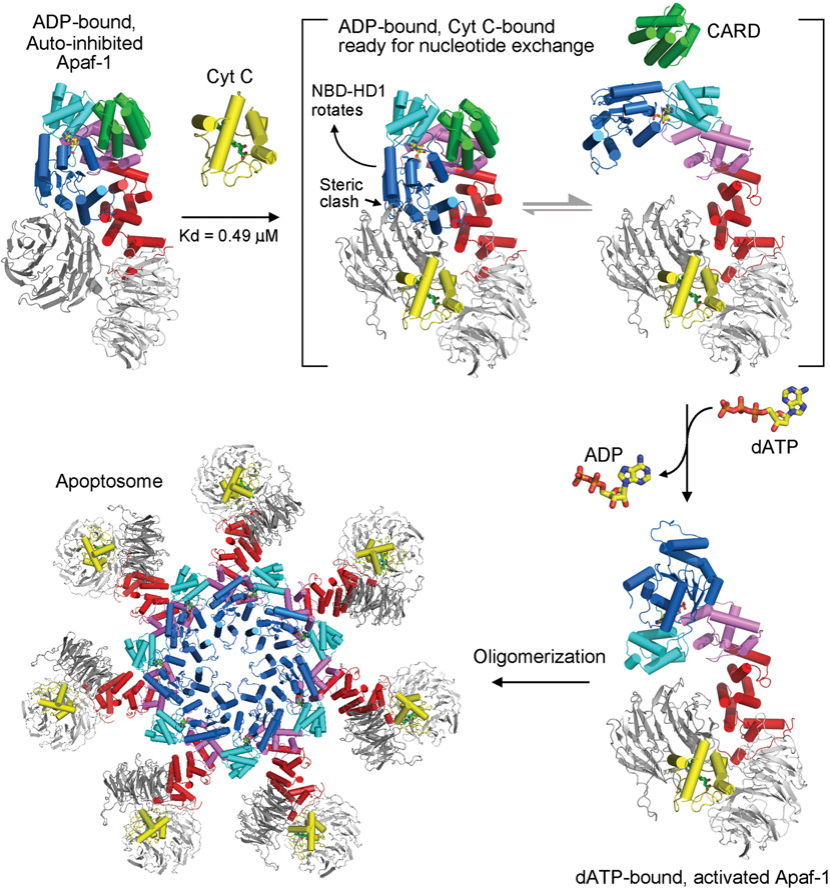
Mechanism of Apaf-1 activation and apoptosome assembly. In the absence of apoptotic stimuli, Apaf-1 exists in cells as an ADP-bound, autoinhibited monomer. At the onset of apoptosis, CytC is released into the cytoplasm, where it binds to the WD2 and WD1 domains of Apaf-1 with a dissociation constant of ∼0.49 µM. CytC binding brings WD1 closer to WD2 and pushes the NBD–HD1 module away, resulting in the departure of WHD from the nucleotide-binding interface and subsequent exposure of the bound ADP to solvent. Such changes may greatly facilitate displacement of ADP by dATP or ATP, which favors the activated conformation of Apaf-1. Seven molecules of activated Apaf-1 assemble into a closed apoptosome.

Our cryo-EM structure, in conjunction with biochemical evidence, unveils the underlying molecular mechanism for the activation of autoinhibited Apaf-1 and the assembly of a functional apoptosome (Fig. 9). Prior to CytC binding, Apaf-1 exists in an autoinhibited, monomeric state, which is characterized by specific interactions between WD1 and the NBD–HD2 domains (Supplemental Fig. S7). In particular, the previously unnoted cation:π interactions between Lys637 of WD1 and Trp249 of NBD are likely to play a major role in locking the autoinhibited conformation of Apaf-1 (Supplemental Fig. S7). In the autoinhibited Apaf-1, the bound ADP molecule is deeply buried and inaccessible to solvent (Fig. 8), which presumably blocks potential nucleotide exchange. CytC binds to Apaf-1 with a dissociation constant of ∼0.49 µM ± 0.06 µM, triggering a 60° rotation of WD1 toward WD2. The conformational switch of WD1 serves two distinct roles: disengaging its interactions with the NBD–HD2 domains and sterically clashing with NBD of the autoinhibited Apaf-1. Thus, CytC binding primes Apaf-1 for nucleotide exchange (Fig. 9). The replacement of ADP by dATP or ATP may stabilize the activated conformation of Apaf-1, thus allowing seven Apaf-1 molecules to assemble into an apoptosome. Compared with the autoinhibited Apaf-1, the WHD–HD2–WD2 rod in the activated Apaf-1 is rotated nearly 180° relative to the NBD–HD1 module.

Our proposed mechanism of Apaf-1 activation and apoptosome formation is consistent with all available structural and biochemical evidence. In this mechanism, the primary role of CytC binding is to destabilize the autoinhibited conformation of Apaf-1 by weakening or disrupting the interactions between WD1 and the NBD–HD2 domains. Truncation of the WD1–WD2 domains from Apaf-1 eliminates these inhibitory interactions, thus allowing the truncated Apaf-1 to form a miniapoptosome that is fully capable of activating caspase-9 (Hu et al. 1998; Srinivasula et al. 1998; Riedl et al. 2005). This mechanism also predicts inefficient or little nucleotide exchange in the absence of CytC binding because the bound ADP molecule is inaccessible to solvent. Although somewhat similar to that proposed by Akey and colleagues (Yuan et al. 2013), our proposed mechanism is based on the 3.8 Å structure of the Apaf-1 apoptosome and structure-guided biochemical analyses and thus contains structurally validated information. For example, the way CytC binds Apaf-1 is different from that proposed previously (Yuan et al. 2013), and the observed interactions are corroborated by biochemical analysis (Figs. 5, 6).

Dark in fruit flies and CED-4 in *C. elegans* are the functional homologs of Apaf-1 in mammals. We previously determined the X-ray structure of the CED-4 apoptosome (Qi et al. 2010) and the cryo-EM structure of the Dark apoptosome (Pang et al. 2015). The advent of the Apaf-1 apoptosome allows comparison of the activated conformations among Dark, CED-4, and Apaf-1 (Supplemental Fig. S8). The Dark protomer can be superimposed to Apaf-1 with a root mean squared deviation (RMSD) of ∼5.95 Å over 260 aligned Cα atoms (Supplemental Fig. S8A). Despite the moderate RMSD value, the four domains NBD, HD1, WHD, and HD2 are reasonably well aligned. However, the WD1 and WD2 domains in Dark are placed much closer to each other than those in Apaf-1 (Supplemental Fig. S8B); this observation is consistent with the notion that CytC is dispensable for assembly of the Dark apoptosome. The CED-4 protomer can be aligned to Apaf-1 with an RMSD value of 2.71 Å over 252 Cα atoms (Supplemental Fig. S8C).

Our structure of the Apaf-1 apoptosome represents a significant step in an age-old quest to understand how caspase-9 is activated. The observed atomic interactions and structural features in the apoptosome not only explain the assembly of apoptosome but also serve as a molecular framework for understanding caspase-9 activation. The CARDs, which are essential for caspase-9 recruitment and activation, are flexible and disordered in the apoptosome. We speculate, as suggested by Akey and colleagues (Yuan et al. 2011), that these CARDs may become ordered and organized upon caspase-9 binding. These yet to be observed features may provide mechanistic insights into apoptosome-mediated activation of caspase-9.

## Materials and methods

### Apaf-1 purification and apoptosome assembly

The full-length human Apaf-1 cDNA with a C-terminal 10xHis tag was cloned in the pFastBac vector, and the baculovirus was generated using the Bac-Bac system (Invitrogen). The recombinant Apaf-1 protein was overexpressed in Hi-5 insect cells (Invitrogen). Forty-eight hours after viral infection, the cells were harvested by centrifugation and homogenized in 20 mM HEPES (pH 7.5), 10 mM KCl, 1.5 mM MgCl_2_, 1 mM EDTA, and 1 mM DTT. Apaf-1 was purified by nickel affinity chromatography (Ni-NTA; Qiagen) and gel filtration (Superose 6, 10/30; GE Healthcare). The apoptosome was assembled by incubating the full-length Apaf-1 with an excess amount of horse CytC (Sigma) at molar ratio of ∼1:2 and 1 mM dATP.

### CytC purification

The untagged, full-length horse CytC was cloned into the pET-Duet vector and coexpressed with the yeast heme lyase CYC3 in *Escherichia coli* BL21 (DE3) cells. All CytC mutants were generated by PCR-based method. The wild-type and mutant CytC proteins were individually purified as previously described (Yu et al. 2001) and used for biochemical analyses.

### Caspase-9 activity assay

The impact of CytC mutations on Apaf-1 assembly was measured by the activity of apoptosome-activated caspase-9 activity. Caspase-3 (C163A) was used as the substrate. The full-length Apaf-1 at 1 μM was incubated with 0.5 μM CytC, 4 µM dATP, 0.05 μM caspase-9, and 14 μM substrate for 60 min at 37°C. The reaction was stopped by adding an equal volume of 2× SDS loading buffer. The cleavage activities were examined by SDS-PAGE and Coomassie staining.

Fluorescent peptide Ac-LEHD-AFC was also used for the detection of caspase-9 protease activity. Caspase-9 at 200 nM, full-length Apaf-1 at 400 nM, and 1 mM dATP were incubated with 400 nM wild-type CytC or mutants for 10 min at 22°C. Next, the fluorescent substrate was added to a final concentration of 200 μM. The fluorescence intensity was monitored in a fluorescence spectrophotometer (F-4600, Hitachi) with an excitation wavelength of 400 nm and an emission wavelength of 505 nm.

### Gel filtration analysis of interactions between Apaf-1 and CytC

The full-length Apaf-1 was individually incubated with different CytC mutants at a molar ratio of 1:2 before loading onto a Superdex-200 column (increase 5/150; GE Healthcare). The column was pre-equilibrated with 100 mM KCl, 20 mM HEPES (pH 7.5), and 5 mM DTT.

### ITC

ITC was used to measure the binding affinity between Apaf-1 and CytC. All proteins were prepared in a buffer containing 100 mM KCl and 20 mM HEPES (pH 7.5). The concentrations of Apaf-1 and CytC were 4 and 60 μM, respectively. The titration was performed at 22°C using a VP-ITC microcalorimeter (MicroCal). Data were fitted using the software Origin 7.0 (MicroCal).

### EM

Three-microliter aliquots of assembled Apaf-1 apoptosome, at a concentration of ∼5 μM, were applied to glow-discharged Quantifoil 400-mesh CuR1.2/1.3 grids. Grids were blotted in a Vitrobot IV (FEI Company) for ∼2–3 sec at 4°C with 100% humidity and then plunge-frozen in liquid ethane. Cryo-EM images of Apaf-1 apoptosome were recorded manually on a K2 Summit detector (Gatan Company) in superresolution mode on an FEI Titan Krios microscope operating at 300 kV. A pixel size of 1.32 Å, defocus values between 1.4 and 3.0 μm, a dose rate of approximately five electrons per square angstrom per second, and an exposure time of 8 sec were used on the K2 Summit detector. UCSFImage4 was used for data collection (developed by Xueming Li).

### Image processing

The 32 movie frames of each micrograph were aligned by whole-image motion correction to correct beam-induced movements (Li et al. 2013). Contrast transfer function parameters of the resulting micrographs were estimated by CTFFIND4 (Mindell and Grigorieff 2003). Using Relion (version 1.4-alpha) (Scheres 2012), 202,932 particles were autopicked from 912 micrographs. After three rounds of reference-free 2D class averaging and one round of 3D classification, 134,919 good particles were selected for 3D refinement. A 40 Å low-pass filtered cryo-EM reconstruction of Apaf-1 apoptosome (The Electron Microscopy Data Bank [EMDB] 5186) (Yuan et al. 2013) was used as an initial model in the 3D refinement. The reconstruction map exhibited an overall resolution of 3.8 Å, with higher resolutions for the central region but relatively poor density for CytC and the two WD40 repeat propellers (WD1 and WD2).

By applying a local mask around one spoke and performing 3D classification without any alignment, particles were classified by only considering the differences in the spoke region, other than the averaged differences from the entire apoptosome. The resulting class with the largest number of particles was chosen for 3D refinement. Only this spoke region and the linked central hub were aligned and refined locally, within 5°. This classification strategy proved to be useful for improving the local map quality. Because an apoptosome has a sevenfold symmetry, we rotated each particle around the symmetry axis seven times to put all seven spokes in the same position for local classification strategy. The original 134,919 particles that had been used to generate the 3.8 Å density map were treated as the first copy. These particles were rotated 360°/7 around the symmetry axis by adding 360/7 to the column “_rlnAngleRot” in the Relion input star file, resulting in the second copy of the particles. The second copy of the particles was similarly rotated by adding another 360/7 to the column “_rlnAngleRot,” generating the third copy of the particles. This operation was repeated four more times to generate the fourth, fifth, sixth, and seventh copies of the particles. For 134,919 particles, 944,433 spokes were rotated to the same position and classified with the local mask. The resulting largest class contained 196,815 spokes in the same configuration. The final reconstruction of these particles markedly improved the resolution in the spoke region to ∼5 Å, into which atomic coordinates from crystals structures were docked. Reported resolutions are based on the gold-standard FSC 0.143 criterion (Chen et al. 2013). The resulting maps from refinement were post-processed by Relion for correction of the modulation transfer function (MTF) of the detector and sharpened by a negative B factor (Rosenthal and Henderson 2003). Local resolution variations were estimated using ResMap (Kucukelbir et al. 2014).

### Model building and refinement

The atomic coordinates of Apaf-1 (Protein Data Bank [PDB] code 3J2T) served as the initial model. The structure was docked into the overall map by COOT (Emsley and Cowtan 2004) and fitted into density by Chimera (Pettersen et al. 2004). Initial structure refinement of Apaf-1 was carried out by Phenix in real space (Adams et al. 2002) with secondary structure and geometry restraints to prevent overfitting. During refinement, sevenfold noncrystallographic symmetry (NCS) restraint was applied. One Apaf-1 protomer was manually adjusted and built using COOT, and the final model was generated with a C7 symmetry operator using the CCP4 program (Collaborative Computational Project 1994). The *Equus caballus* CytC (PDB code 4RSZ) was docked into the improved density of this region from the local masking map.

### Accession numbers

The atomic coordinates of the Apaf-1 apoptosome have been deposited in the PDB with the accession code 3JBT. The EM map has been deposited in the EMDB with accession code EMD-6480.

## Supplementary Material

Supplemental Material
