# Supplementary material for: Atomic structure of the apoptosome: mechanism of cytochrome c- and dATP-mediated activation of Apaf-1
Source: Genes Dev. 2015 Nov 15;29(22):2349–61. doi: 10.1101/gad.272278.115 (PMC4691890; doi:10.1101/gad.272278.115)
Supplement: Supplemental Material [file supp_29_22_2349__index.html]

Atomic structure of the apoptosome: mechanism of cytochrome c- and dATP-mediated activation of Apaf-1 — Supplemental Material 

# Atomic structure of the apoptosome: mechanism of cytochrome *c*- and dATP-mediated activation of Apaf-1

## Supplemental Material

**Files in this Data Supplement:**

- Supp Material.pdf
