# Supplementary material for: Atomic structure of the apoptosome: mechanism of cytochrome c- and dATP-mediated activation of Apaf-1
Source: Genes Dev. 2015 Nov 15;29(22):2349–61. doi: 10.1101/gad.272278.115 (PMC4691890; doi:10.1101/gad.272278.115)
Supplement: Supplemental Material [file supp_gad.272278.115_SuppMaterials.pdf]

SUPPLEMENTAL MATERIAL

Supplemental Table S1: Cryo-EM data collection and model statistics.

Supplemental Figure S1: Cryo-EM analysis of the Apaf-1 apoptosome.

Supplemental Figure S2: Representative EM density maps.

Supplemental Figure S3: Overall structure of the Apaf-1 apoptosome.

Supplemental Figure S4: The top face of the apoptosome contains a strip of positively charged amino acids (lower left panel) whereas the bottom face is enriched by negatively charged residues (lower right panel).

Supplemental Figure S5: Comparison of CytC binding in the two dATP-bound, activated Apaf-1 protomers derived from our structure (color green) and a previous report (grey).

Supplemental Figure S6. Assessment of the abilities of five CytC mutants to interact with Apf-1.

Supplemental Figure S7: Interactions between WD1 and the NBD-HD2 domains may lock Apaf-1 in an auto-inhibited state.

Supplemental Figure S8: Structural comparison among the activated Dark, CED-4, and Apaf-1 molecules.

Supplemental Table S1. Cryo-EM data collection and model statistics.

**Data collection**	
EM equipment	FEI Titan Krios
Voltage (kV)	300
Detector	Gatan K2
Pixel size (Å)	1.32
Electron dose (e-/Å2)	40
Defocus range (μm)	1.4~3.0
**Reconstruction**	
Software	RELION 1.4α
Number of used Particles	134,919
Accuracy of rotation (˚)	1.073
Accuracy of translation (pixels)	0.784
Final Resolution (Å)	3.8
**Model composition**	
Protein residues	8736
dATP	7
Mg	7
Heme	7
Fe	7
**Validation**	
R.m.s deviations	
Bonds length (Å)	0.012
Bonds Angle (˚)	1.402
Ramachandran plot statistics (%)	
Most favored	82.5
Additional allowed	13.3
Generously allowed	3.0
Disallowed	1.2

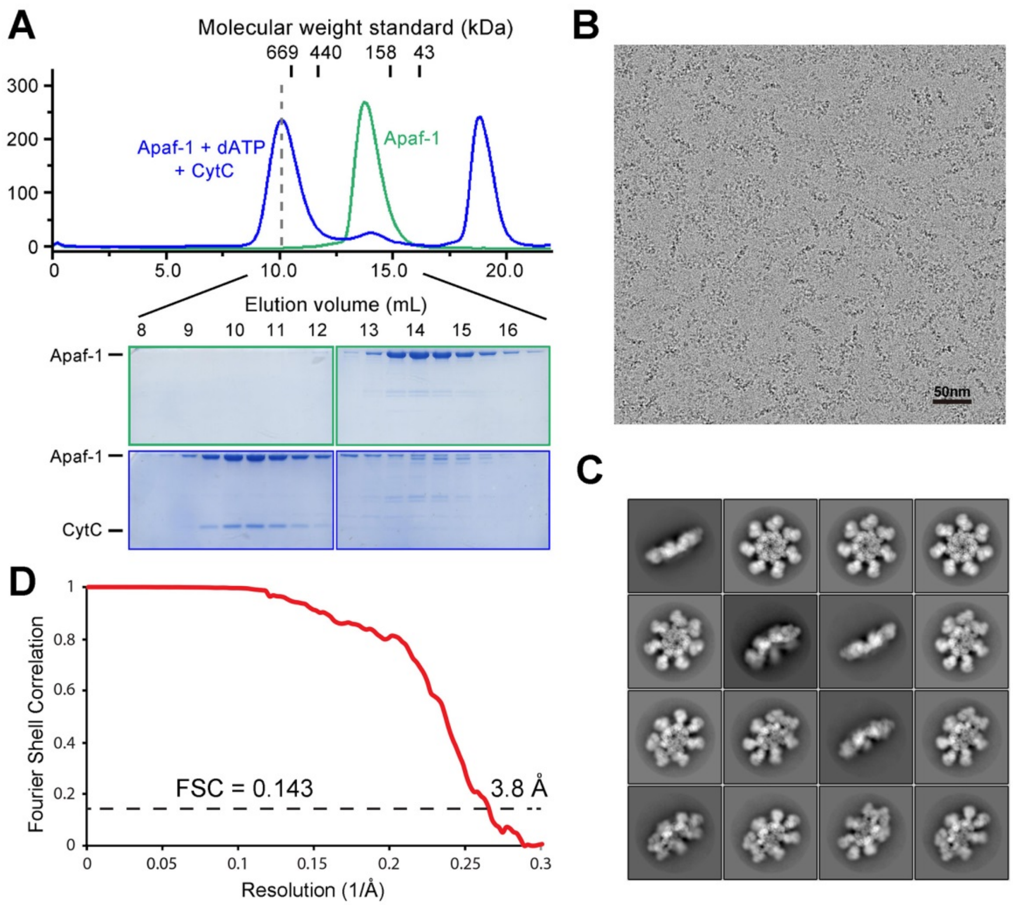

Supplemental Figure S1. Cryo-EM analysis of the Apaf-1 apoptosome. (A) Apaf-1 assembles into an apoptosome in the presence of cytochrome *c* (CytC) and dATP. Shown here are gel filtration chromatograms (upper panel) and SDS-PAGE gels stained by Coomassie blue (lower panels). (B) A representative micrograph of the cryo-EM sample of the Apaf-1 apoptosome. (C) Representative 2D class averages of the Apaf-1 apoptosome. (D) The overall resolution of the cryo-EM structure is 3.8 Å on the basis of the gold FSC standard.

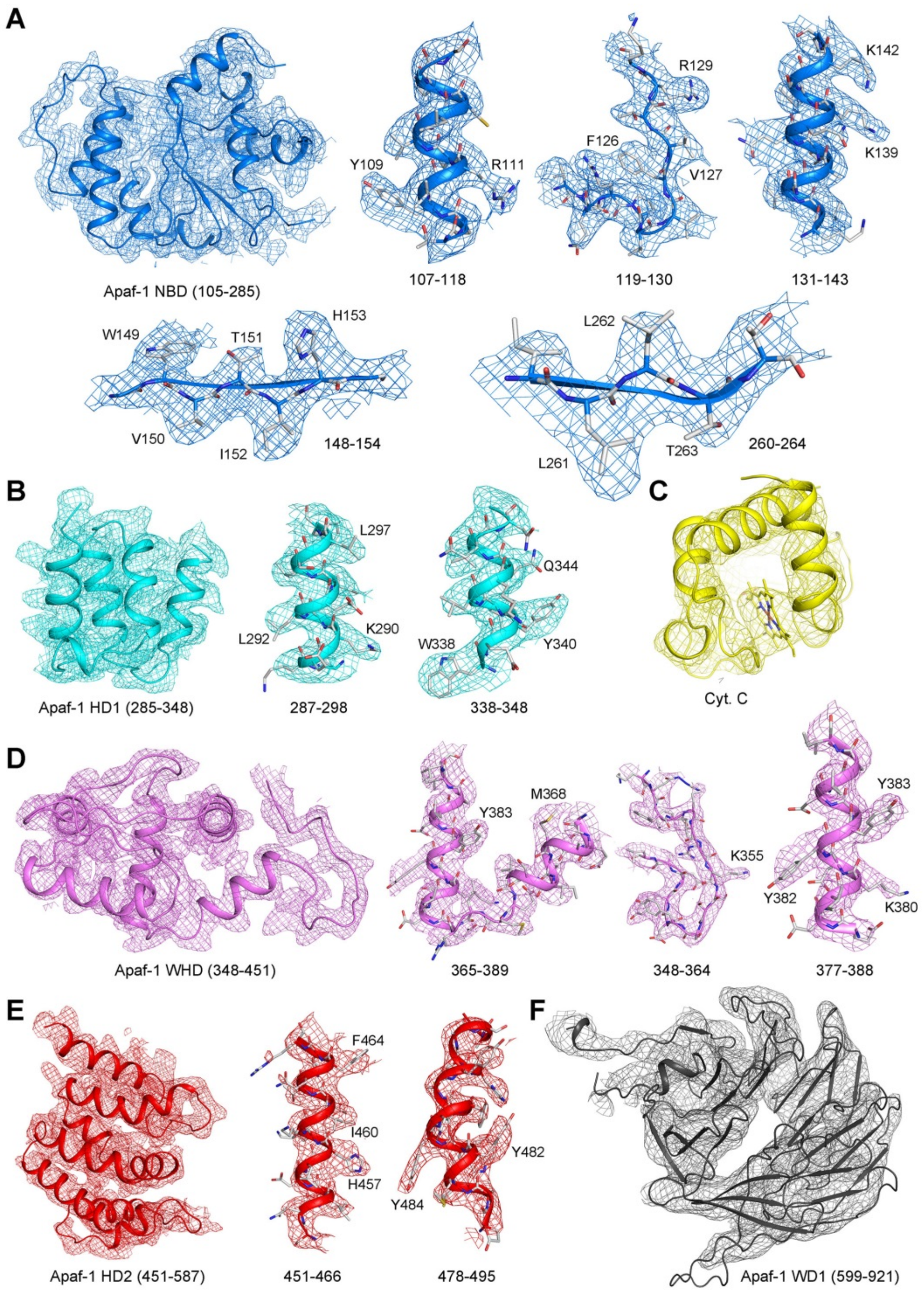

Supplemental Figure S2. Representative EM density maps. (A) Representative EM density in the NBD domain. Some of the key residues with clear EM density are labeled. (B) Representative EM density in the HD1 domain. (C) The overall EM density of CytC. (D) Representative EM density in the WHD domain. (E) Representative EM density in the HD2 domain. (F) Representative EM density in the WD1 domain.

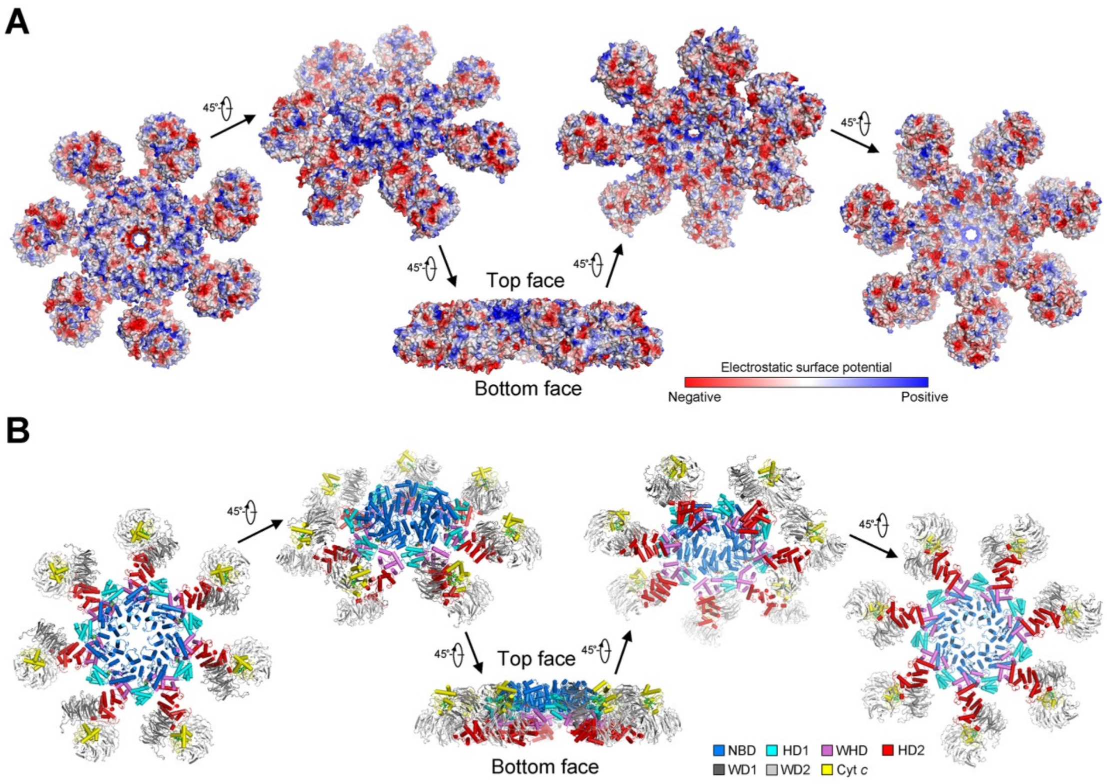

Supplemental Figure S3. Overall structure of the Apaf-1 apoptosome. (A) Structure of the apoptosome is displayed by electrostatic surface potential. Five successive views are shown. (B) Structure of the apoptosome is shown in cartoon representation.

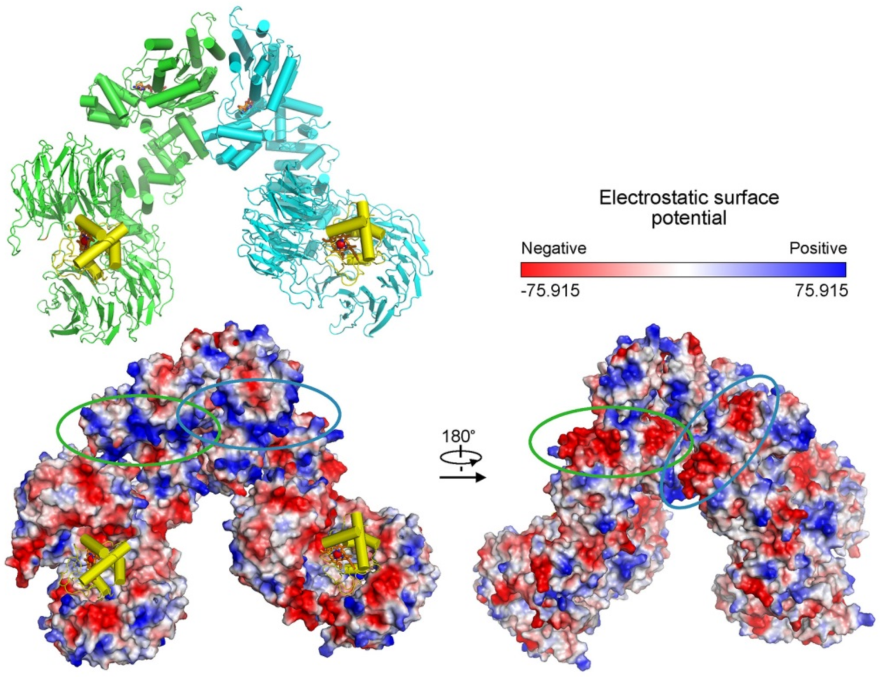

Supplemental Figure S4. The top face of the apoptosome contains a strip of positively charged amino acids (lower left panel) whereas the bottom face is enriched by negatively charged residues (lower right panel).

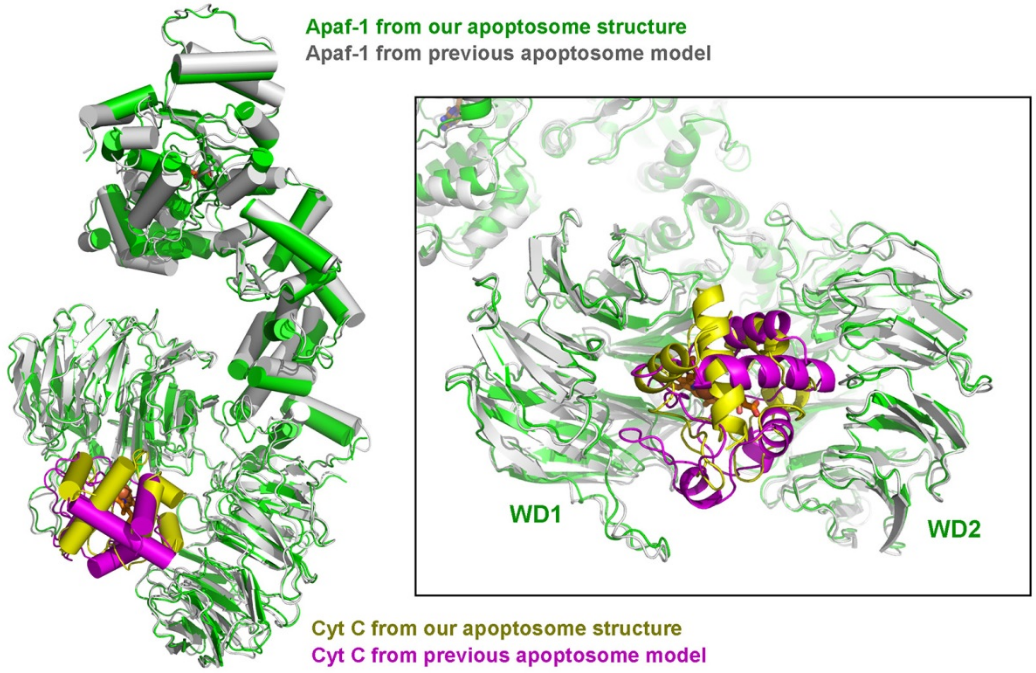

Supplemental Figure S5. Comparison of CytC binding in the two dATP-bound, activated Apaf-1 protomers derived from our structure (color green) and a previous report (grey). These two Apaf-1 molecules can be aligned to each other with an RMSD value of 1.68 Å over 1103 Cα atoms. Notably, the way CytC is recognized is quite different in these two cases.

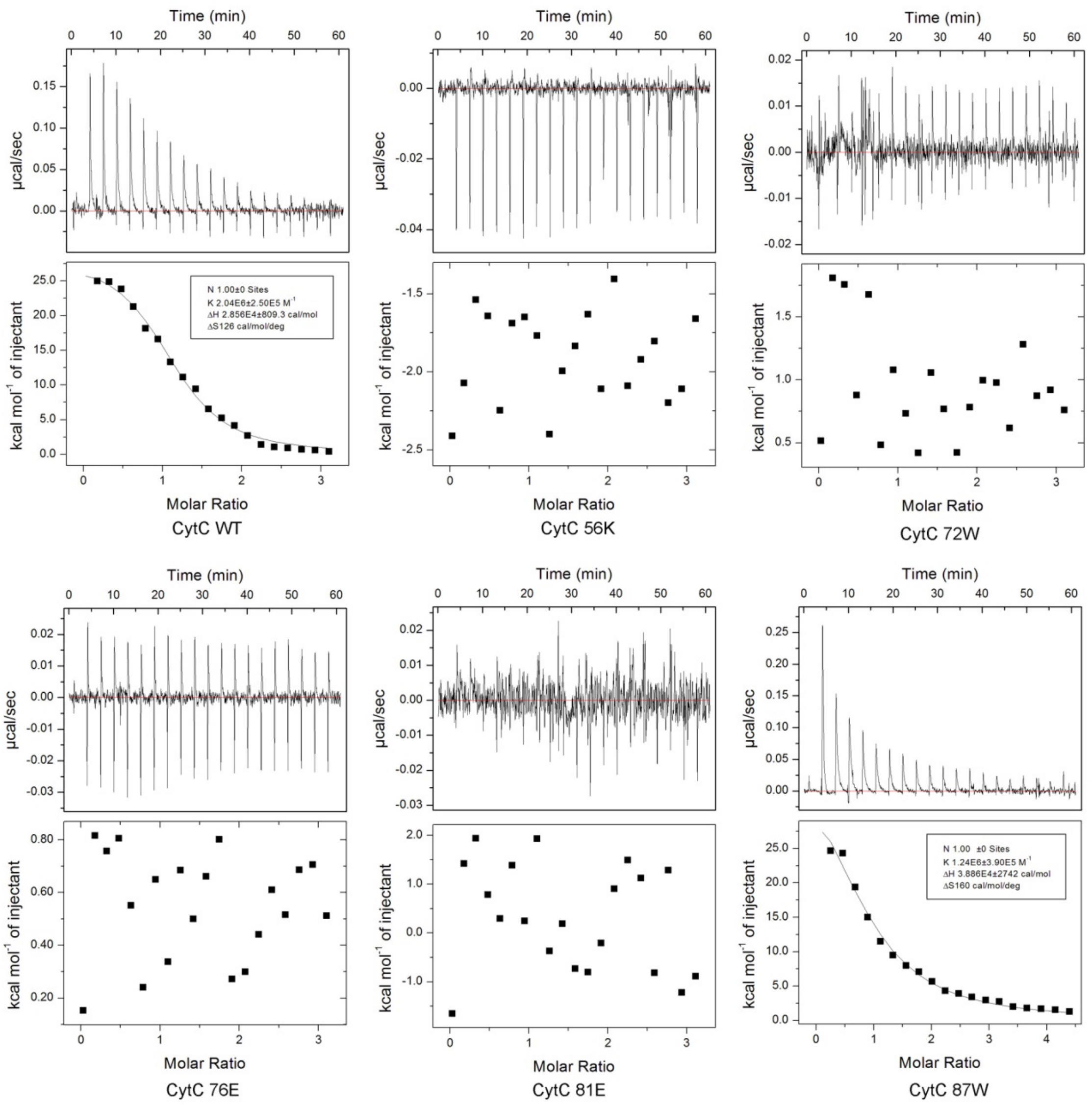

Supplemental Figure S6. Assessment of the abilities of five CytC mutants to interact with Apf-1. Shown here are ITC titration curves and curve fittings for WT and five CytC mutants. For each CytC variant, the raw ITC titration data and the fitted curve are shown in the upper and lower panels, respectively.

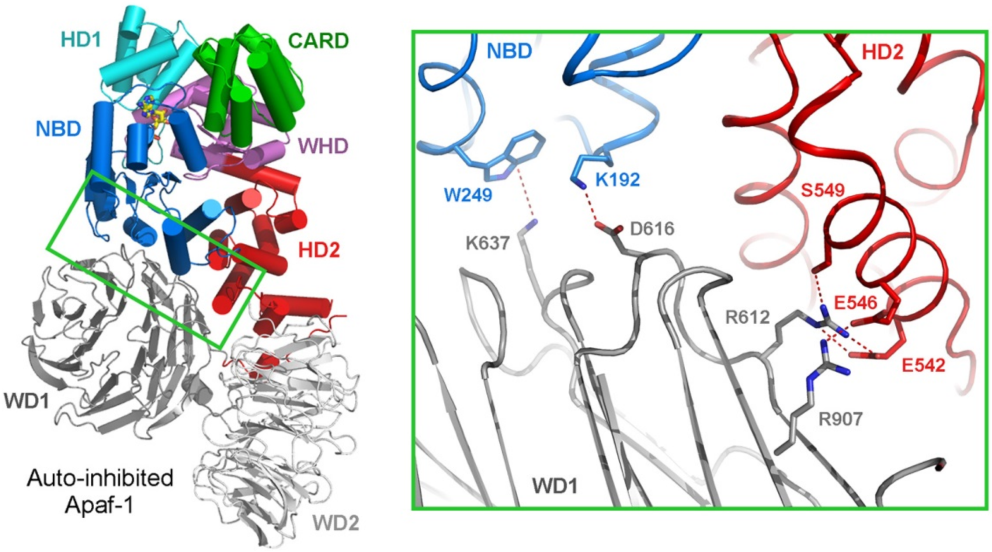

Supplemental Figure S7. Interactions between WD1 and the NBD-HD2 domains may lock Apaf-1 in an auto-inhibited state. Of these interactions, the cation:π interaction between Lys637 from WD1 and Trp249 from NBD might be particularly important.

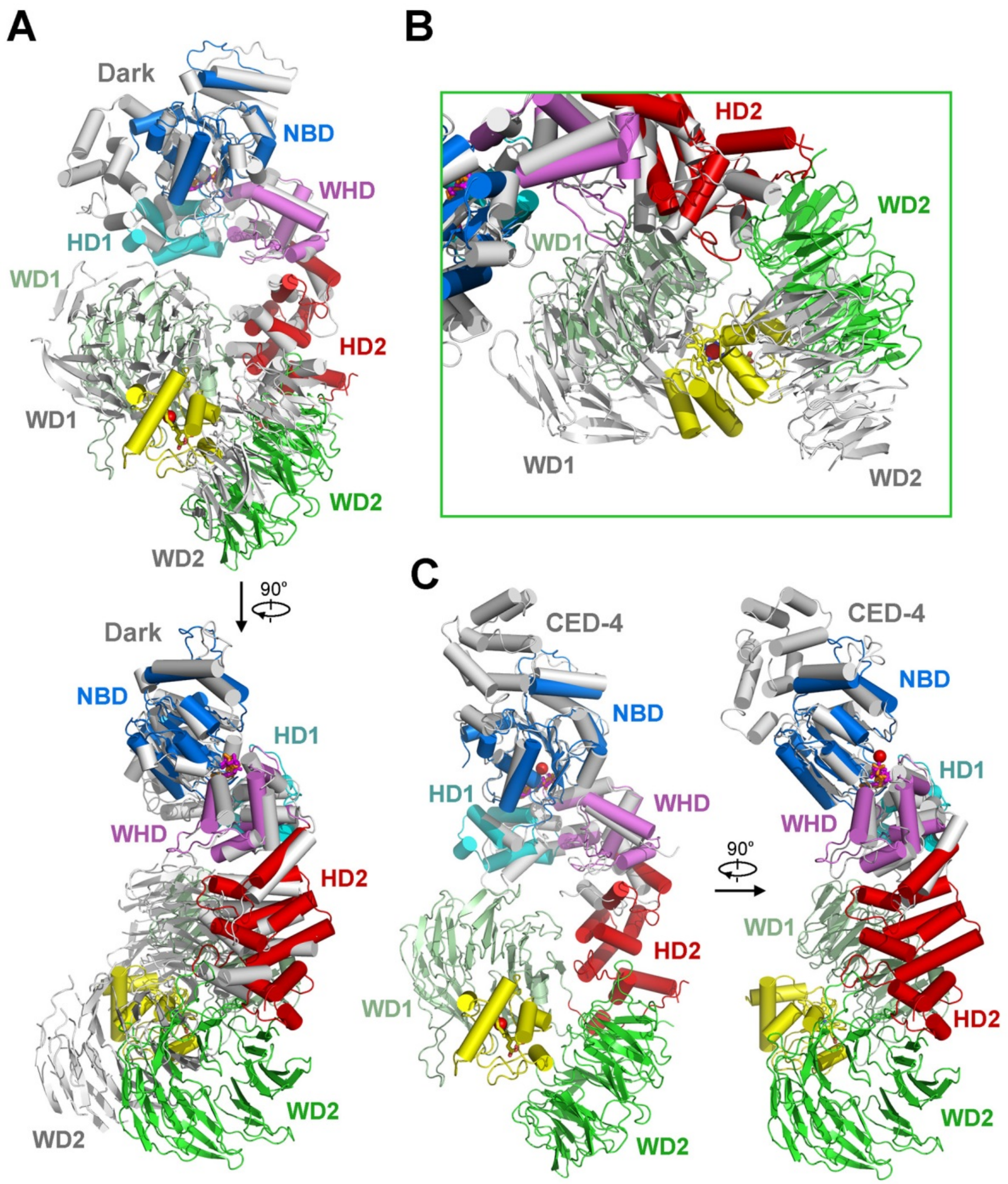

Supplemental Figure S8. Structural comparison among activated Dark, CED-4, and Apaf-1 molecules. (A) Structural alignment between Dark (grey) and Apaf-1. Two perpendicular views are shown. The various domains in Apaf-1 are color-coded. (B) A close-up view on the CytC binding region. The two WD propellers in Dark are too close to accommodate a CytC molecule. (C) Structural alignment between CED-4 (grey) and Apaf-1. Two perpendicular views are shown.
